# Strain Mediated Voltage Control of Magnetic Anisotropy and Magnetization Reversal in Bismuth-Substituted Yttrium Iron Garnet Films and Mesostructures

**DOI:** 10.1021/acsami.5c14761

**Published:** 2025-11-21

**Authors:** Walid Al Misba, Miela J. Gross, Kensuke Hayashi, Daniel B. Gopman, Caroline A. Ross, Jayasimha Atulasimha

**Affiliations:** † Mechanical and Nuclear Engineering, 6889 Virginia Commonwealth University, Richmond, Virginia 23220, United States; ‡ 2167 Electrical Engineering and Computer Science, Massachusetts Institute of Technology, Cambridge, Massachusetts 02139, United States; § Department of Materials Science and Engineering, Massachusetts Institute of Technology, Cambridge, Massachusetts 02139, United States; ∥ Department of Materials Physics, Graduate School of Engineering, Nagoya University, Nagoya 464-8603, Japan; ⊥ Materials Science & Engineering Division, 10833 National Institute of Standards and Technology, Gaithersburg, Maryland 20899, United States; # Electrical and Computer Engineering, Virginia Commonwealth University, Richmond, Virginia 23284-3043, United States; ¶ Department of Physics, Virginia Commonwealth University, Richmond, Virginia 23284-3043, United States

**Keywords:** magnetic anisotropy modulation, Bi-YIG films, substrate, voltage-induced strain, voltage control, magnetization

## Abstract

We report on magnetic anisotropy modulation in Bismuth-substituted Yttrium Iron Garnet (Bi-YIG) thin films and mesoscale patterned structures deposited on a PMN–PT substrate with the application of a voltage-induced strain. The Bi content is selected for low coercivity and higher magnetostriction than that of YIG, yielding significant changes in the hysteresis loops through the magnetoelastic effect. The piezoelectric substrate is poled along its thickness, which is the [011] direction, by applying a voltage across the PMN–PT/SiO_2_/Bi-YIG/Pt heterostructure. In situ magneto-optical Kerr effect microscopy (MOKE) shows the modulation of magnetic anisotropy with voltage-induced strain. Furthermore, voltage control of the magnetic domain state of the Bi-YIG film at a fixed magnetic field produces 90° switching of the magnetization easy axis above a threshold voltage. The magnetoelectric coefficient of the heterostructure is 1.05 × 10^–7^ s m^–1^ which is competitive with that of other ferromagnetic oxide films on ferroelectric substrates such as La_0.67_Sr_0.33_MnO_3_/PMN–PT and YIG/PMN–PZT. Voltage control of magnetization reversal fields in 5–30 μm wide dots and racetracks of Bi-YIG show potential for energy-efficient nonvolatile memory and neuromorphic computing devices.

## Graphical abstract

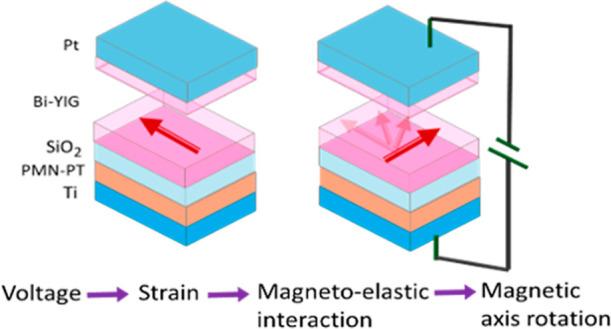

## 1 Introduction

Electric field tunability of magnetization is particularly appealing for high density magnetic memory with lower energy consumption^1–3^ compared to current-controlled technologies.^4,5^ In this regard, multiferroic structures with coupled ferroelectric (FE) and ferromagnetic properties have been examined for their ability to control the electric and magnetic ordering simultaneously through the converse magnetoelectric effect (CME).^6,7^ The required electric current density to write a magnetic random-access memory bit is on the order of 10^11^ A/m^2^ with 10 fJ^8^ dissipation compared to 1–100 aJ dissipation in capacitive multiferroic devices.^8,9^ Although single phase multiferroic materials^10,11^ are the most direct embodiment of this phenomenon, composite heterostructures provide three to four orders of magnitude greater magnetoelectric coupling as well as stability of both polarization and magnetization at room temperature.^12,13^ Several mechanisms have been explored for harnessing CME from composite heterostructures, such as transferring mechanical strain from the FE to the ferromagnet,^14–17^ modulation of the spin-up and spin-down densities of states at the FE–ferromagnet interface,^18^ and modification of an oxide ferromagnet through voltage-driven oxygen migration.^19^ Strain transfer mechanisms demonstrate low heat dissipation per switching cycle and high magnetoelectric coupling coefficients.^20^

Relaxor ferroelectric materials such as (Pb (Mg_1/3_Nb_2/3_)­O_3_)_1–*x*_–(PbTiO_3_)_*x*_ (PMN–PT) show large piezoelectric coefficients when operated near the morphotropic phase boundary (*x* = 0.3, for PMN–PT) and have been employed to transfer strain to a ferromagnetic material.^21^ Thin films of ferromagnetic materials with low to moderate magnetostriction such as Ni,^22^ Co,^14,15,23,24^ CoFeB,^16,25^ or FeGa^26^ have been grown on top of PMN–PT to investigate magnetoelectric effects. The magnetic films in these composites are often amorphous or polycrystalline, enabling electric-field-induced magnetoelastic anisotropy to exceed magnetocrystalline anisotropy for 90° rotation of the magnetic easy axis. Complete 180° switching was demonstrated in patterned Co/PMN–PT by sequentially applying voltages in the electrode pairs.^27^

In contrast to ferromagnets, many ferrimagnetic oxides offer more efficient and faster control of magnetization due to their low damping and moderate saturation magnetization. Ferrimagnetic oxides such as yttrium iron garnet (YIG) and rare-earth iron garnets (REIGs) have been used to demonstrate spin wave propagation and spin torque phenomena.^28,29^ In addition, saturation magnetization, magnetostriction, anisotropy, and Gilbert damping parameter can be modified by inserting rare earth ions.^30^ Despite these advantages, the growth of ferrimagnetic garnets on piezoelectric compounds poses a significant challenge due to lattice incompatibility, thus limiting the potential to harness the benefit of electrical control. We developed a thin film processing strategy centered around the growth of a thin, amorphous SiO_2_ buffer that enables growth of high-quality polycrystalline ferrimagnetic garnets on bulk piezoelectric substrates.^31^ This materials processing advance allowed us to evaluate strain-induced anisotropy modulation of yttrium-substituted dysprosium iron garnet (Y-DyIG) film crystallized on PMN–PT,^31^ when the PMN–PT was poled.

Bi-YIG has certain advantages over other REIGs due to its low loss tangent,^32^ large domain wall velocities,^33^ low Gilbert damping,^34,35^ and magneto-optical activity^36^ making it important to many applications in magneto-optics such as optical isolators,^37^ electrical current sensors,^38^ spintronics,^39^ and magnonic devices such as spin wave carriers.^40^ In addition, Bi-YIG is an insulator that minimizes electron collision events resulting in reduced energy dissipation at high frequencies compared to conductors. The nonconductive nature presents an opportunity for high-frequency applications to obviate lamination architectures typically required by metallic magnetoelastic materials. Moreover, magnetoelectric bilayers consisting of a magnetic garnet and a piezoelectric material bonded together are reported;^41,42^ they are not suitable for scaling down to implement in a device. In this study, we demonstrate magnetoelectric control of Bi-YIG insulator thin films that are deposited on PMN–PT and patterned into microstructures, going beyond macroscopically bonded bilayers of magnetic garnets and piezoelectric materials. In situ magneto-optical Kerr microscopy (MOKE) studies show electrical-field control of magnetization and electrically tunable magnetic properties. We show that the magnetic easy axis of Bi-YIG films can be reoriented by 90° under an applied electric field. Furthermore, we show voltage control of magnetization reversal of micrometer-sized magnetic dots and racetracks. These results suggest a role for Bi-YIG in energy-efficient voltage-controlled memory and neuromorphic devices.^44,45^

## 2 Sample Growth and Characterization Methods

Following process conditions used for DyIG,^31^ a series of 0.5 mm thick, (011)-oriented PMN–PT [(PbMg_0.33_Nb_0.67_O_3_)_1–*x*_(PbTiO_3_)_*x*_; *x* = 0.29–0.33] substrates were coated with an amorphous, 2.4 nm thick SiO_2_ buffer layer by radio frequency magnetron sputtering. The 45.6 nm thick Bi-YIG films were grown on the PMN–PT/SiO_2_ heterostructures in addition to fused silica (SiO_2_) and (100)-oriented Si substrates using pulsed laser deposition (PLD) at room temperature by codeposition from stoichiometric YIG (Y_3_Fe_5_O_12_) and BFO (BiFeO_3_) targets to yield a composition of Bi_2.13_Y_1.40_Fe_5_O_*x*_.^46^ This high-Bi composition has a higher magnetostriction which counteracts the in-plane shape anisotropy in tensile-strained films and can even lead to an out-of-plane easy axis in Bi-YIG on fused silica.^46^ A 248 nm KrF excimer laser was used at an energy of 600 mJ and a repetition rate of 10 Hz and was focused to a fluence of about 2 J cm^–2^ at each target. The laser shots on each target were adjusted based on the calibrated growth rates. The chamber was pumped to a base pressure of 1.33 mPa (1 × 10^–5^ Torr), and an oxygen pressure of 2.7 Pa (20 mTorr) was maintained during the deposition. The films then underwent ex situ annealing in a furnace for 72 h at 600 °C in order to crystallize the garnet (more details in Supporting Information Section S5).

Grazing incidence X-ray diffraction (GIXD) and film thickness X-ray reflectivity (XRR) measurements were performed using a Rigaku Smartlab Multipurpose Diffractometer with a Cu Kα X-ray source.^43^ Magnetic hysteresis curves of the films were measured using a Digital Measurements Systems Vibrating Sample Magnetometer Model 1660 with a field applied both within the plane and normal to the plane of the substrate. A Zeiss Merlin high-resolution scanning electron microscope (SEM) was used to examine the grain structure. Films were patterned into ellipses and tracks with a minimum dimension of 5 μm by photolithography using a direct-write Heidelberg uMLA exposure system and dry etching using an ion beam etch system.

Figure 1 summarizes the structural and magnetic properties of the Bi-YIG film grown on SiO_2_-buffered PMN–PT. GIXD is sensitive to the diffraction peaks from the film and shows a set of peaks characteristic of polycrystalline garnet, Figure 1a, without a strong texture. Consistent with previous observations,^34,46,47^ the film possesses a preferred magnetization direction within the plane, a saturation magnetization of 101 ± 5 kA/m, and an in-plane coercivity of 10 ± 5 mT. Bi-YIG grown on Si under the same conditions had a magnetoelastic anisotropy of 6 kJ/m^3^,^48^ and the magnetostriction is interpolated as −2.6 × 10^–6^ implying an in-plane tensile stress of the order 1.5 GPa. The Bi-YIG/PMN–PT is expected to have a slightly lower stress state than Bi-YIG/Si based on the thermal expansion of PMN–PT^49^ vs Si. Although magnetoelastic anisotropy promotes PMA, shape anisotropy is dominant, and the film has an in-plane easy axis. SEM micrographs are presented for the Bi-YIG deposited on Si and PMN–PT/SiO_2_ in Figure 2. A low area fraction of small amorphous regions is observed within the predominantly crystallized specimen over the extent of the examined region. Grain sizes are mainly in the range of 1–3 μm and some show a radiating pattern characteristic of low angle grain boundaries that develop as the grains grow.^50^ Crystallization of Bi-YIG over a SiO_2_ amorphous buffer layer avoids epitaxy with PMN–PT, which would yield an orthoferrite-structured film instead of garnet. A thinner buffer than 2.4 nm can have discontinuities and pinholes which would promote orthoferrite formation where the film contacts the substrate. In this experiment, the 2.4 nm buffer successfully inhibited orthoferrite formation. The Bi-YIG film is much thicker, and the silica has little effect on strain transfer to the garnet from the piezoelectric substrate. This buffer layer technique can be extended to other garnet compositions and substrate materials, enabling integration with, for example, amorphous dielectric layers within semiconductor chips.

**Figure 1:**
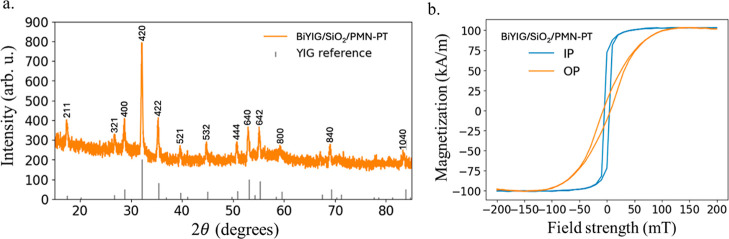
(a) GIXD diffraction image shows Bi-YIG growth on the SiO_2_/PMN–PT substrate. Data has been shifted vertically for clarity. Reference powder diffraction peaks for YIG are indicated. (b) Hysteresis loops taken via vibrating sample magnetometry of the BiYIG/SiO_2_/PMN–PT sample. The curves were measured with the field applied out of plane (OP) and in plane (IP) to the sample surface.

**Figure 2:**
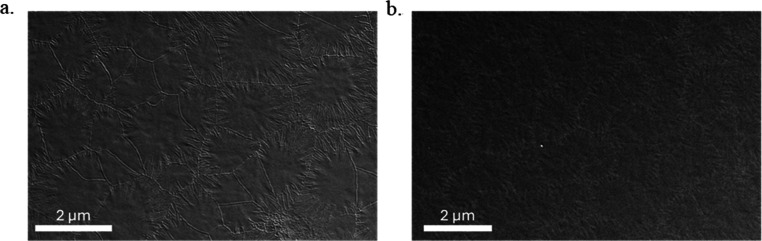
Top surface SEM images of (a) Si/Bi-YIG and (b) PMN–PT/SiO_2_/Bi-YIG.

## 3 Magnetic Hysteresis Modulation with Strain

The magnetic properties of the ferromagnetic material in an FE–ferromagnet heterostructure can be modulated by utilizing the piezoelectric properties of the FE crystal. Applying a voltage across the thickness of the PMN–PT (i.e., along the film normal, defined as *ẑ*, the [011] direction) generates an electric field *E* leading to a piezoelectric strain of different signs in the FE-crystal along the two orthogonal in-plane directions, *x̂*, the [100] and *ŷ*, the [011̅] direction as shown in the heterostructure schematic in Figure 3a. When the electric field is zero, Bi-YIG shows isotropic magnetic behavior within the plane. An electric field along *ẑ* leads to compressive strain along *x̂* and tensile strain along *ŷ* of the substrate, breaking the degeneracy of the Bi-YIG in-plane hysteresis loops.

**Figure 3:**
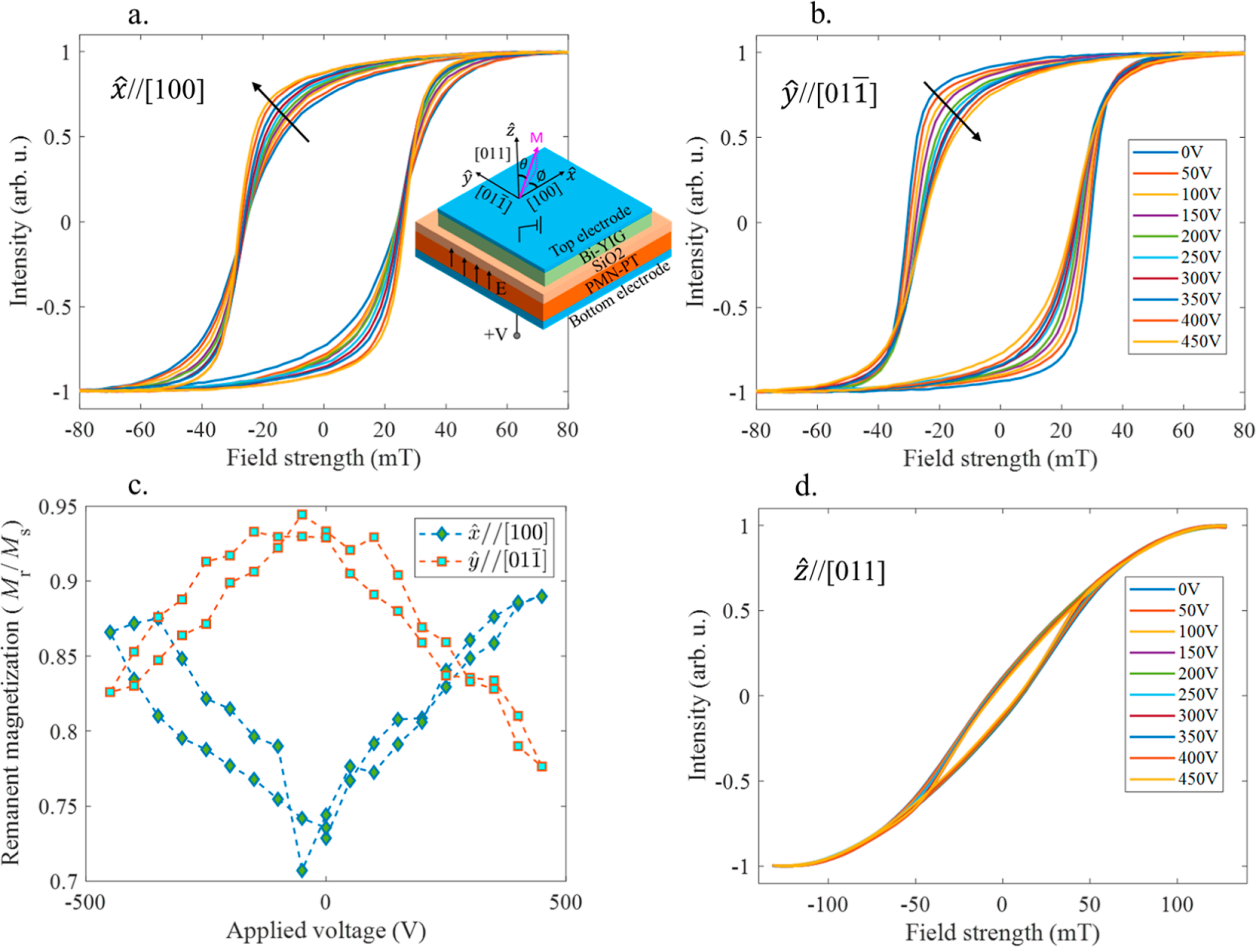
(a,b) Hysteresis loops obtained from MOKE magnetometry for different voltages applied along the thickness of the heterostructure, PMN–PT/SiO_2_/Bi-YIG when the magnetic field is applied along the in-plane direction (a) *x̂* and (b) *ŷ*. Black arrows indicate the trend for increasing voltage. The inset in (a) shows a schematic of the heterostructure with the direction of the applied voltage, principal axes, and the polar angle, θ and azimuthal angle, φ of the BiYIG film magnetization, M. (c) Ratio of remanent and saturation magnetization vs the applied voltage for both in-plane directions, *x̂* and *ŷ*. (d) Hysteresis loops as a function of voltage obtained from polar MOKE for the out-of-plane direction, *ẑ*.

To characterize the magnetoelectric behavior of the composite, we first poled the PMN–PT/SiO_2_/Bi-YIG by applying 450 V along *ẑ* (*E* = 0.9 MV/m) for 90 min and then set the voltage to zero. We then applied voltages in 50 V increments, capturing in-plane hysteresis loops using the in situ longitudinal MOKE magnetometry signal at each voltage, as shown in Figure 3a,b. Blue light (wavelength ≈465 nm) was used because Bi-YIG has a high MOKE response at this wavelength. The as-deposited sample in Figure 1b is isotropic in plane and showed similar magnetic hysteresis loops along *x̂* and *ŷ*.^31^ Poling and subsequent relaxation lead to a remanent strain in the PMN–PT, which is tensile along *x̂* and compressive along *ŷ*.^31,51^ Hence, after poling and relaxation to 0 V, the Bi-YIG shows a harder magnetization direction (lower remanence and squareness) along *x̂* and an easier direction along *ŷ* (see hysteresis loops in Figure 3a,b at 0 V) compared to the as-grown state, consistent with a negative magnetostriction.^52,53^ When voltages ranging from 50 to 450 V are subsequently applied to the poled sample, the PMN–PT experiences increasing compressive strain along *x̂* due to the negative piezoelectric coefficient, *d*_31_ of PMN–PT,^51^ and tensile strain along *ŷ* due to the positive piezoelectric coefficient, *d*_32_ of PMN–PT (see hysteresis loops in Figure 3a,b from 50 to 450 V). This leads to an anisotropy reorientation in the Bi-YIG with *x̂* becoming the easy in-plane direction for a sufficiently large voltage.

With respect to θ and φ, the polar and azimuthal angles of magnetization shown in Figure 3a, the magnetoelastic energy can be expressed as

$$F_{me}=-\frac{3}{2}\lambda_{s}\frac{Y}{1+\vartheta }\varepsilon_{xx}\sin^{2}\theta \cos^{2}\varphi -\frac{3}{2}\lambda_{s}\frac{Y}{1+\vartheta }\varepsilon_{yy}\;{\;\sin}^{2}\;\theta \sin^{2}\varphi$$

where ε_*xx*_ and ε_*yy*_ are the strains along *x̂* and *ŷ*, *Y* is the Young’s modulus, λ_s_ is the saturation magnetostriction, and ϑ is the Poisson’s ratio of Bi-YIG. There is no stress along *ẑ* due to the free boundary condition at the top surface. The saturation magnetostriction coefficient of polycrystalline Bi-YIG is negative, from which it follows that the magnetoelastic free energy is reduced when the magnetization is aligned along a compressively strained direction. An estimate of the lower bound of λ_s_ may be calculated from the change in magnetoelastic energy density, $K_{\mathrm{x̂}\mathrm{x̂}}$ (calculated using the hysteresis loop area, details in Supporting Information Section S3) due to the change in applied voltage, Δ*V* as $\lambda_{S}=\frac{2\times \left(1+\vartheta \right)\times t_{PMN-PT}\times \Delta K_{\mathrm{x̂}\mathrm{x̂}}}{3Y\times d_{31}\times \Delta V}\approx -3.6\times 10^{-6}$ , which is consistent with the interpolated value of −2.6 × 10^–6^. Here, *Y* = 200 GPa is the Young’s modulus of Bi-YIG film, ϑ = 0.3 is the Poisson ratio,^31^ *d*_31_ = −900 pC/N,^51^ and *t*_PMN–PT_ = 0.5 mm is the thickness of the PMN–PT.

Figure 3a shows the hysteresis loops become increasingly square along *x̂* as the voltage is increased from 0 to 450 V, an indication of the development of a magnetic easy axis along that direction. The coercive field increases from 25 ± 2 mT at 0 V to 27 ± 2 mT at 450 V, and the saturation field decreases from 77 ± 2 mT at 0 V to 57 ± 2 mT at 450 V. Opposite trends are observed along *ŷ*: the loop becomes less square with increasing voltage, and the coercivity decreases from 30 mT ± 2 mT at 0 V to 24 mT ± 2 mT at 450 V (Figure 3b). A high squareness ratio, defined as *Sq* = *M*_r_/*M*_s_ where *M*_r_ and *M*_s_ are the remanent and saturation magnetization, respectively, indicates the easy axis. *Sq* increases (decreases) along *x̂* (*ŷ*) with an increasing voltage (Figure 3a,b). A butterfly-like hysteresis loop is observed for *M*_r_ vs *V* (Figure 3c) which illustrates the magnetoelectric coupling between the applied voltage and remanent magnetization. The loop measured along the poling direction, *ẑ*, using polar MOKE shows little change with voltage (Figure 3d). We did not observe any significant asymmetry (horizontal shift) in the MOKE curves in Figure 3a,b,d. The average asymmetry observed in Figure 3a,b is 1.3 and 1.2 mT, respectively, which is within the measurement uncertainty of the external magnetic field, ±2 mT (magnetic field step size used to measure the hysteresis loops). As there are no interfaces that could lead to exchange bias, we attribute the asymmetry to the measurement uncertainty.

The anisotropy constants $K_{eff,\mathrm{x̂}ŷ}$ , $K_{eff,\mathrm{x̂}ẑ}$ , and $K_{eff,ŷẑ}$ under different electric fields are estimated by first scaling the hysteresis loops in Figure 3a,b,d with saturation magnetization *M*_s_ and then calculating the hysteresis loop areas (average of the descending and ascending branches) after separating the anhysteric components.^54^ The details are presented in Section S3. The estimated $K_{eff,\mathrm{x̂}ŷ}$ at 0 V is computed to be 840 ± 80 J/m^3^ which decreases with increasing voltages and becomes −450 ± 45 J/m^3^ at 450 V. This suggests the longitudinal direction *x̂* becomes easier for magnetization as we increase the voltage and vice versa for direction *ŷ*.

In Figure 4, we analyze the magnetization switching of the poled heterostructure for two cases, 0 V and 450 V for the in-plane directions *x̂* and *ŷ*. Initially, a reference background image is taken, from which the images acquired at different magnetic fields are subtracted. At positive saturation, +89 mT, predominantly white contrast domains are observed. The images in Figure 4b,d correspond to the positions marked on the hysteresis loops in Figure 4a,c, respectively.

**Figure 4:**
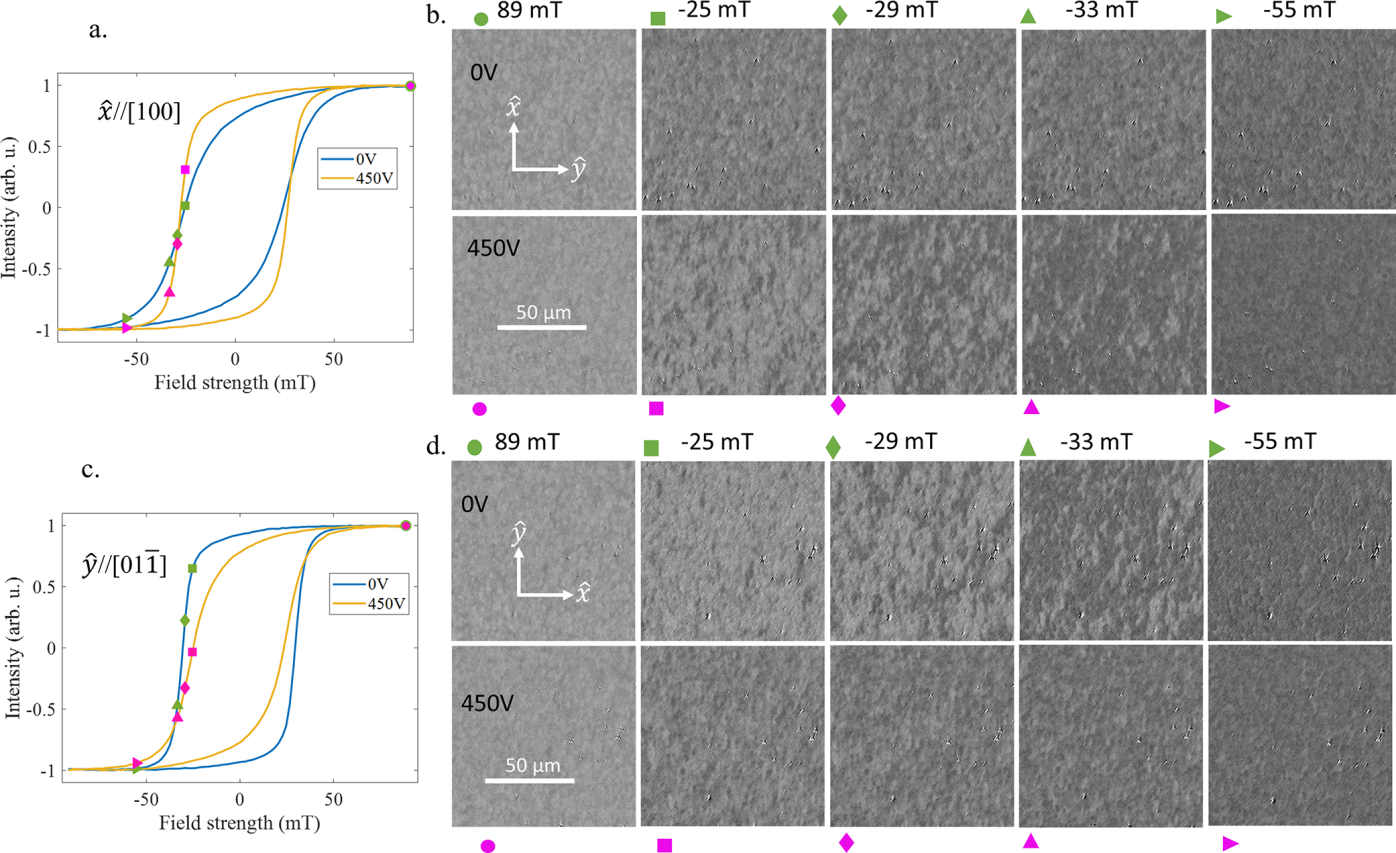
(a) Hysteresis curves with external fields applied along the in-plane direction *x̂* when the heterostructure is subjected to an applied voltage of 0 and 450 V. (b) Longitudinal MOKE images showing the magnetization reversal process. The corresponding field values for which the images are taken are also marked in the hysteresis loops using green and purple polygon markers. (c) Hysteresis loops for in-plane direction *ŷ* for 0 and 450 V and (d) corresponding magnetization reversal images.

As the external field is increased in the negative direction, a reversal is indicated by the black contrast. For fields applied along *x̂*, when the voltage is 0 V, the switching corresponds to a gradual change in contrast, and no significant domain wall propagation is observed along the hard *x̂* axis (compare the images at −29 mT and −33 mT in bottom panels of Figure 4b which corresponds to an easy *x̂* and therefore a sharper transition with more prominent domain wall nucleation and propagation features). Along the *ŷ* direction, the easy axis switching process occurs with domain wall nucleation and propagation at 0 V, but a gradual contrast change is observed at 450 V, consistent with *ŷ* becoming a hard axis with increasing voltage.

## 4 Magnetization Reversal with the Electric Field

Figure 5 shows the voltage control of magnetic domains at a fixed magnetic field. The sample was first saturated by applying a field of −70 mT, then the field was set to +27 mT, and the corresponding domain patterns were observed as a function of voltage. The field of +27 mT was selected because it is close to the coercive field for both of the in-plane directions and led to significant changes in the domain pattern with voltage. To cycle the electric field, the sample was first poled by applying 450 V and subsequently relaxed to 0 V. For image acquisition in the *x̂* direction, 450 V is applied and followed by a magnetic field of −70 mT while the voltage is maintained at 450 V. In this configuration, domains with black contrast are predominant. The external field is then increased to +27 mT. White-contrast domains indicate the onset of reversal, which increases as the voltage is reduced stepwise in increments of 50 V, from 450 to 0 V, while keeping the magnetic field constant at 27 mT. The domain pattern shows little change for voltages below 100 V. The behavior is explained by the *x̂* axis being the easy axis at 450 V but becoming less easy as the voltage decreases until it becomes the hard axis by 0 V. The reduction in anisotropy along the *x̂* axis leads to magnetization rotation toward the *ŷ* axis, reduction in domain sizes, and a weakening of contrast. At low voltages, *x̂* becomes a magnetically hard direction and the magnetization orientation within the domains is governed by the balance between the magnetoelastic anisotropy and the Zeeman energy. An analogous but opposite trend is found when the field is applied along *ŷ*, shown in Figure S1 of the Supporting Information. Thus, in Figure 5, the preferred axis of magnetization shifts from *ŷ* to *x̂* as the voltage is increased from 0 to 450 V and a 90° switching of the easy axis is accomplished. Angular dependent hysteresis loops are measured to further illustrate the 90° switching of the magnetization easy axis with the application of voltage. The experimental details are presented in Section S4. We note that we performed at least two trials for the experiments, as shown in Figures 5 and 4, and the results matched qualitatively. The variation comes from a slightly different bias field or a different location on the sample on which the MOKE is performed with the 50× objective.

**Figure 5:**
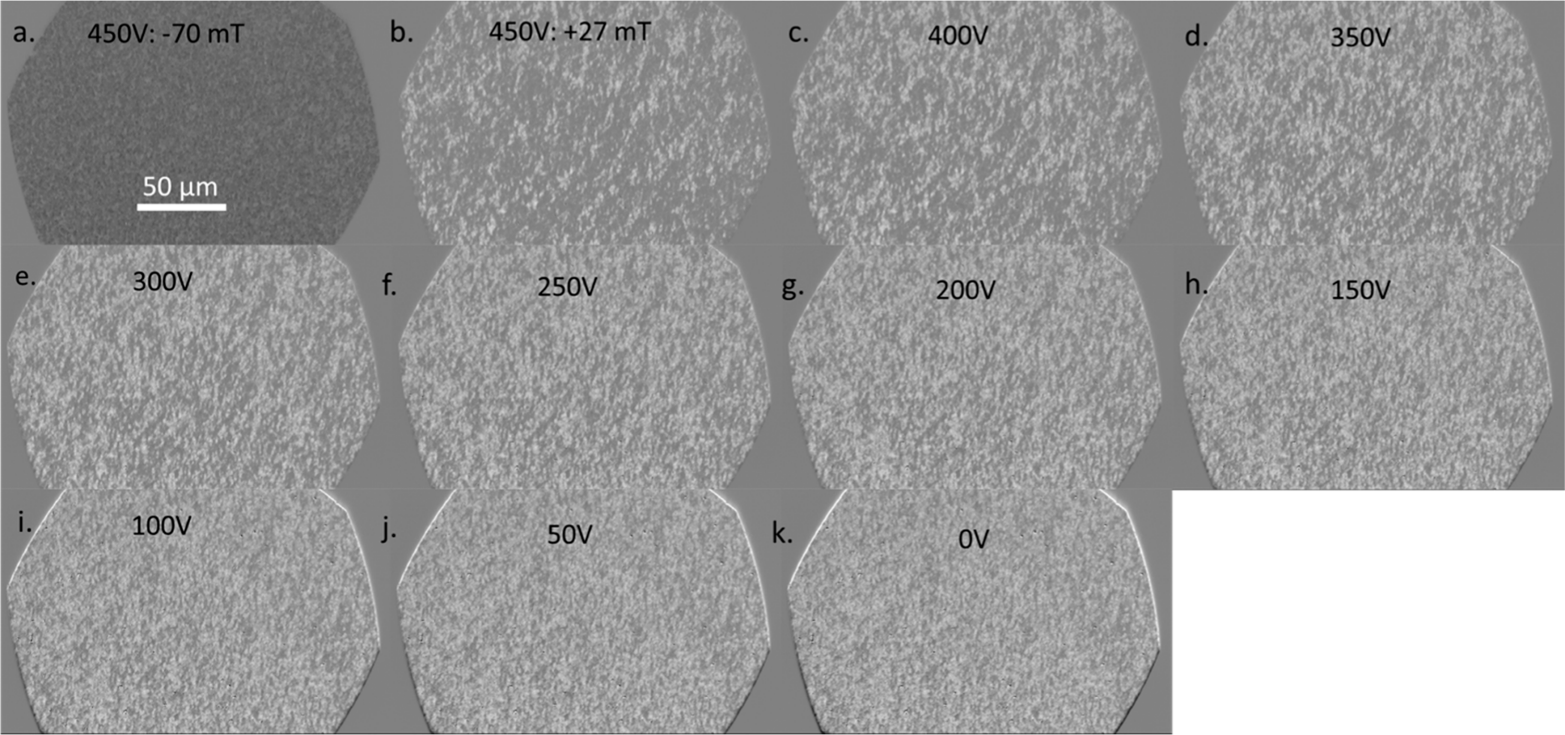
MOKE images showing the saturated domains and reversal of the domains for varying amplitude voltages at a fixed reversal field along the in-plane direction *x̂*//[100]. (a) The sample is poled at 450 V and saturated with a −70 mT field. The external field is then fixed at +27 mT, while the voltage remains at (b) 450 V and decreases to (c) 400 V, (d) 350 V, (e) 300 V, (f) 250 V, (g) 200 V, (h) 150 V, (i) 100 V, (j) 50 V, and (k) 0 V.

The magnetoelectric coefficient, $\alpha_{E}=\mu_{0}\frac{\Delta M}{\Delta E}$ ^55^ of the PMN–PT/SiO_2_/Bi-YIG is calculated from Figure 3c (in-plane direction, *x̂*), under the condition of zero applied field. Thus, Δ*M* is the change in the remanent magnetization, Δ*M*_r_ and, $\Delta E=\frac{\Delta V}{t}$ , where *t* is the thickness of the heterostructure, *E* is the electric field, and *V* is the applied voltage across the heterostructure. The highest value of α_E_ is determined to be 1.05 × 10^–7^ s m^–1^, which is achieved when the voltage is changed from 0 V to −50 V with a maximum change in remanent magnetization of ≈8.36 kA/m. Table 1 compares the ME coefficient of PMN–PT/SiO_2_/Bi-YIG (this work) with those of other ferroelectric/magnetic bilayers. Table 1 shows that the estimated magnetoelectric coefficient of PMN–PT/SiO_2_/Bi-YIG is comparable with previously examined ferroelectric/magnetic oxide systems, though lower than ferroelectric/magnetic metal systems.

**Table 1:** Comparison of Magnetoelectric Coefficient

ferroelectric/-magnetic materials	magnetic film type	magnetoelectric coefficient (s m^–1^)
PMN–PT/Bi-YIG	oxide	1.05 × 10^–7^ [this work]
PMN–PT/Y-DyIG	oxide	2.8 × 10^–7^ ^31^
PMN–PT/YIG	oxide	5.4 × 10^–9^ ^58^
PMN–PZT/YIG	oxide	1.8 × 10^–7^ ^59^
PMN–PT/La_0.7_Sr_0.3_ MnO_3_	oxide	6.4 × 10^–8^ ^60^
PMN–PT/FeGa	metallic	2.7 × 10^–6^ ^61^
PMN–PT/Co_2_FeSi	metallic	1 × 10^–5^ ^62^
BaTiO_3_/FeRh	metallic	1.6 × 10^–5^ ^63^

To compare the dynamical properties of polycrystalline Bi-YIG with that measured on single crystal films, we measured the ferromagnetic resonance of the fused silica/Bi-YIG sample at frequencies between 5 and 9 GHz (Figure S2). The frequency range is too small to determine damping, but the line widths were approximately 200 mT compared with as low as 0.4–5 mT for single crystal epitaxial YIG and Bi-YIG grown on a garnet substrate.^34,35,52,56^ The higher line width is attributed to the anisotropy distribution arising from the polycrystalline microstructure, the residual amorphous nonmagnetic regions, and the high film stress^46,57^ and can likely be reduced by optimizing the process or composition. Getting low damping polycrystalline Bi-YIG is indeed a challenge, but we recently got a significant reduction in line width (factor of 4) by growing YIG on a yttria-stabilized zirconia substrate, which has a better thermal expansion match to YIG than Si and other substrates.

Finally, to check the reproducibility of the strain-dependent hysteresis of PMN–PT/SiO_2_/Bi-YIG, we investigated another sample with 55 nm Bi-YIG thickness. This sample shows a similar trend in strain-induced anisotropy modulation and a magnetoelectric coefficient of 0.9 × 10^–7^s m^–1^.

## 5 Voltage Control of Magnetism in Bi-YIG Microstructures

The effect of the electric field is studied on PMN–PT/SiO_2_/Bi-YIG microstructures fabricated using photolithography and dry etching. Patterned ellipses and racetracks were examined to see the effect of the magnetoelectric coupling on voltage control of magnetization switching and domain evolution. Figure 6 presents the magnetization switching in ellipses when different voltages are applied across the microstructures. The easy axes of the ellipses are parallel to the in-plane substrate direction *x̂*. The samples are first poled at 450 V and then relaxed. The ellipses are saturated with a −20 mT field applied along *x̂*, and the field is increased to 20 mT in 0.5 mT intervals to observe the magnetization evolution during switching. The magnetization in the ellipses is switched at a lower field along the *x̂* direction when the voltage is modified from 450 to 0 V. For example, the ellipses are mostly switched from the negative *x̂* direction (black contrast) to the positive *x̂* direction (white contrast) at a 8 mT field for 0 V compared to cases of 450 V where the bigger ellipse is not even switched at 10 mT. Thus, the switching field of the patterned magnets can be controlled by modifying the electric field across the PMN–PT substrate similarly to the Bi-YIG films. This can be explained as follows. As we observe in Figure 3a, the bulk Bi-YIG films show high coercivity values along the *x̂* direction at *V* = 450 V. The value decreases as we reduce the voltage to 0 V. Similarly, in the elliptical dots, higher external magnetic fields (due to high coercivity) are required to achieve complete magnetization reversal at 450 V. Although the coercivity trend is similar, much lower saturation fields and coercive fields are observed in the microstructures than in the bulk films. We note that when varying voltages are applied at a fixed magnetic field as in Figure 5, the effective field due to voltage-induced strain is small and does not change the magnetic texture significantly as it needs to overcome pinning. This is why we used a fixed voltage to change the anisotropy and applied magnetic fields that are sufficient to overcome pinning and move domains while the constant (fixed) voltage changes the anisotropy to sufficiently influence the average fields at which the domains nucleate, move, and reverse.

**Figure 6:**
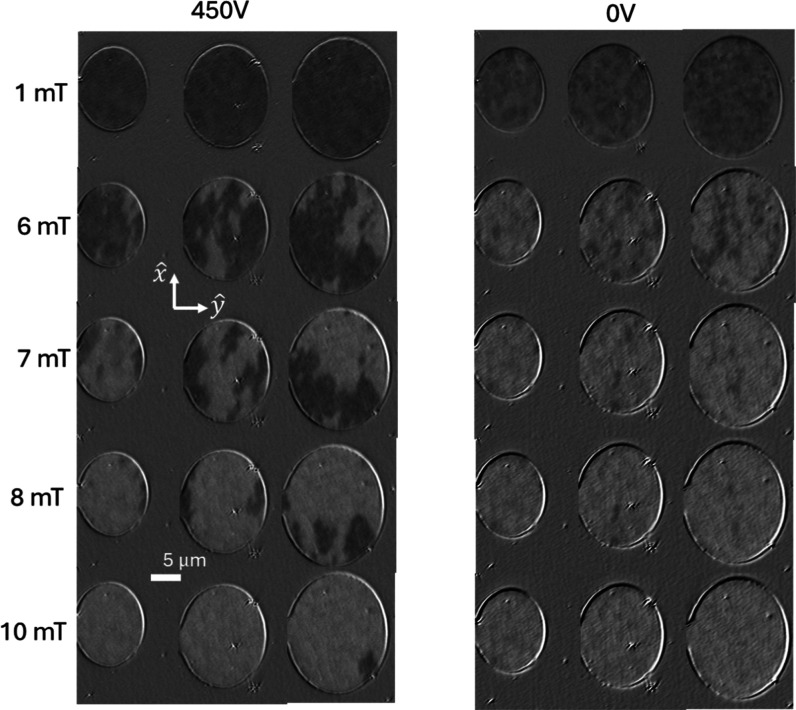
Snapshots of magnetization switching in elliptical microscale magnets of Bi-YIG patterned on PMN–PT for two different voltages. The magnetic fields are applied along in-plane direction *x̂* which is parallel to the easy axes of the ellipses. The elliptical magnets switched at a lower field (8 mT) for *V* = 0 V compared to *V* = 450 V.

Next, the effect of the electric field was studied in racetracks with their long axis parallel to the *x̂* direction. Domain wall nucleation and propagation are observed due to the field applied along the *ŷ* direction. Both the racetrack and the nucleation pads were saturated to −30 mT and the field was increased to +30 mT in 1 mT increments. The corresponding MOKE images are presented in Figure 7 for the poled sample under two different voltages, *V* = 0 V and *V* = 450 V. With the increase in voltage, the nucleation field of the domain wall decreases. Greater movement of domain walls from the pad to racetrack is observed at 7 mT for 0 V. However, the domain wall propagation becomes easier at 450 V and starts to propagate with as little as a 6 mT field. Electric field-induced modulation of the propagation field of the domain wall in the Bi-YIG racetrack can be utilized to work as a synaptic element or spintronic neuron of a neural network. Recent studies show that ferromagnetic racetracks with only a few stable domain wall positions can map the neural network weights (emulate synaptic functionalities) when the racetrack devices are arranged in crossbar architectures.^45,64,65^ Ferrimagnetic racetracks may provide faster, lower-power operation, and the voltage-controlled domain wall propagation may be able to emulate the functionality of a neuron in a spiking neural network,^66^ where the neurons are only activated by applying voltages above a certain threshold.

**Figure 7:**
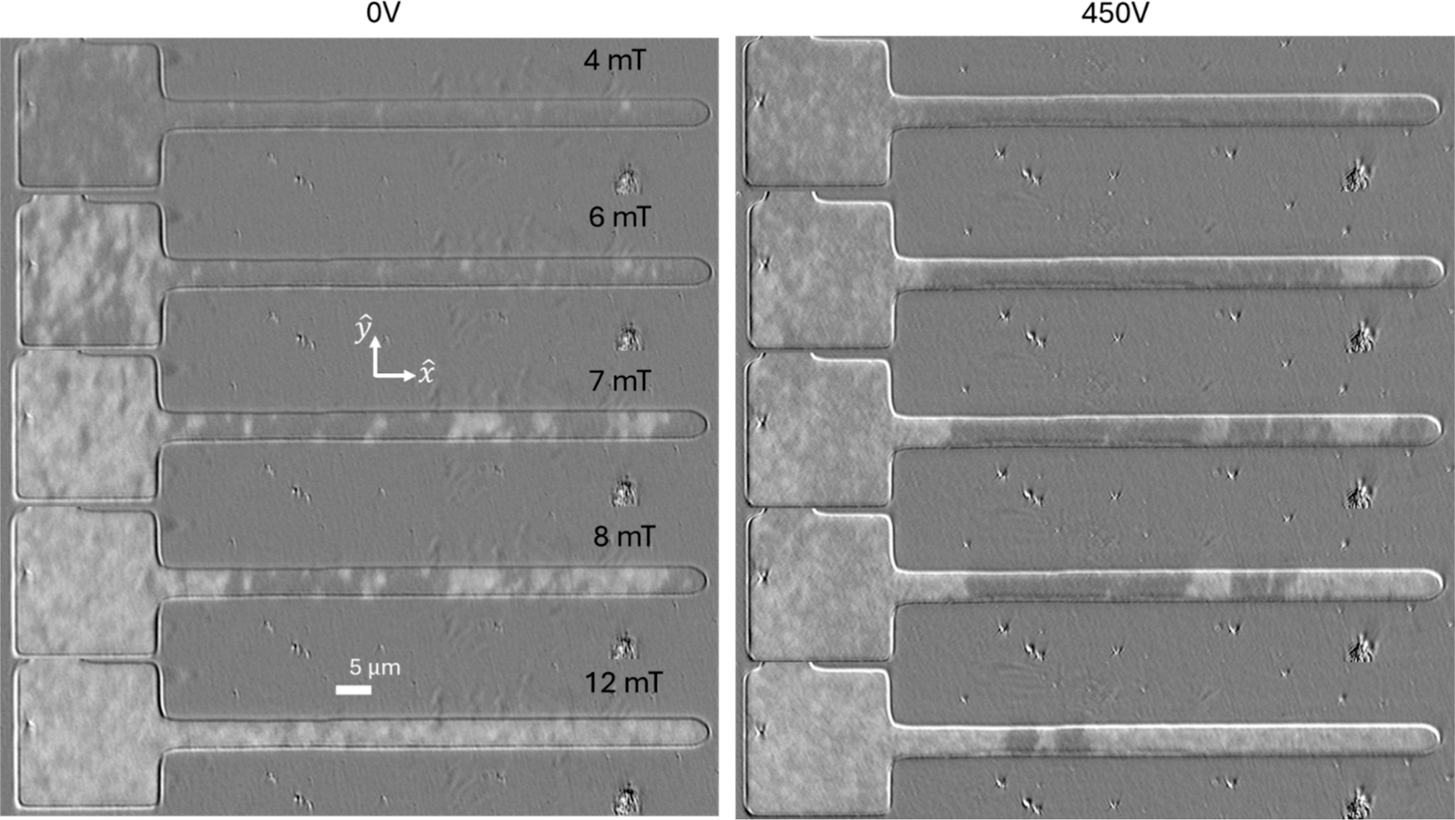
Snapshots of domain wall nucleation and propagation in 5 μm wide racetracks of Bi-YIG patterned on PMN–PT for two different voltages. The magnetic fields are applied along in-plane direction *ŷ*. The domain wall nucleated in the pads propagates along the racetracks at 6 mT when it is subjected to a voltage of 450 V.

## 6 Summary and Conclusion

In summary, we have shown 90° switching of the magnetization easy axis of a multiferroic heterostructure, PMN–PT/SiO_2_/Bi-YIG by using a voltage-induced strain. The Bi-YIG film was fabricated using pulsed laser deposition, and the ratio of Bi and YIG was selected for a high Bi content. An intermediate SiO_2_ buffer layer is deposited between PMN–PT and Bi-YIG to avoid growth of perovskite phases and thereby facilitate garnet crystallization. MOKE magnetometry shows domain wall nucleation and propagation in Bi-YIG films with the application of electric fields and strain-mediated voltage control of magnetization reversal fields in patterned mesostructures. Although the magnetoelectric coefficient is moderate compared to heterostructures combining magnetostrictive metals with PMN–PT, it compares well with other oxide ferrimagnet/PMN–PT structures. The magnetoelectric response can stimulate novel devices that use resonant effects^67–69^ and lead to energy-efficient magnetic memory and neuromorphic computing devices.^70,71^

## Supplementary Material



## Notes

Domain reversal study using magneto-optical Kerr effect microscopy for in-plane direction *ŷ*//[011̅], ferromagnetic resonance, estimation of anisotropy coefficient, angular dependent hysteresis loops, and Bi-substituted YIG (Bi-YIG) (PDF)
