# Supplementary material for: Strain Mediated Voltage Control of Magnetic Anisotropy and Magnetization Reversal in Bismuth-Substituted Yttrium Iron Garnet Films and Mesostructures
Source: ACS Appl Mater Interfaces. 2025 Nov 21;17(48):65946–55. doi: 10.1021/acsami.5c14761 (PMC12679540; doi:10.1021/acsami.5c14761)
Supplement: Supplementary file 1 [file am5c14761_si_001.pdf]

Supplemental Information for

Strain mediated voltage control of Magnetic Anisotropy and magnetization reversal in Bismuth Substituted Yttrium Iron Garnet films and meso-structures

Walid Al Misba [1], Miela J. Gross [2,3], Kensuke Hayashi [3,4], Daniel B. Gopman [5], Caroline A. Ross [3], Jayasimha Atulasimha [1,6,7]

[1] Mechanical and Nuclear Engineering, Virginia Commonwealth University, Richmond, VA, USA
[2] Electrical Engineering and Computer Science, Massachusetts Institute of Technology, Cambridge, MA, USA
[3] Department of Materials Science and Engineering, Massachusetts Institute of Technology, Cambridge, MA, USA
[4] Department of Materials Physics, Graduate School of Engineering, Nagoya University, Nagoya, Japan
[5] Materials Science & Engineering Division, National Institute of Standards and Technology, Gaithersburg, MD, USA
[6] Electrical and Computer Engineering, Virginia Commonwealth University, Richmond, VA, USA
[7] Department of Physics, Virginia Commonwealth University, Richmond, VA, USA

S1. Domain Reversal Study using Magneto-optical Kerr Effect (MOKE) Microscopy for In-Plane Direction y // [011]:

The sample is first poled by applying 450 V and subsequently relaxed to 0 V. The voltage is kept at 0 V and the sample is saturated by applying an external field of -70 mT along the in-plane direction y. Domains with black contrast are prominent and occupy the observed regions as can be seen in Fig. S1a. The reversal field is then set to +27 mT and MOKE images are acquired by increasing the voltages in steps of 50 V up to 450 V. With the application of voltage, white contrast reserved domains are nucleated across the observed regions and the number of reversed domains increases with voltage. In the poled sample, at 0 V, y -axis is the easy axis of magnetization due to the remnant compressive strain from the PMN-PT substrate [S1, S2]. This is evident from the square-shaped hysteresis loops as in Fig. 4c (in the main manuscript) and the prominent domain wall nucleation and growth in Fig. 4d at 0 V. Due to the higher magnetic anisotropy and coercive field (30 mT $\pm$ 2 mT), fewer numbers of reversed domains are seen for +27 mT field at 0 V. As we increase the voltage, the y -direction becomes harder for the magnetization due to the tensile strain from PMN-PT, thus the coercive field decreases. Correspondingly, the quantity of reversed domains increases. Significant changes in the quantity of nucleated domains are not observed beyond a bias of 400 V or higher across the PMN-PT substrate.

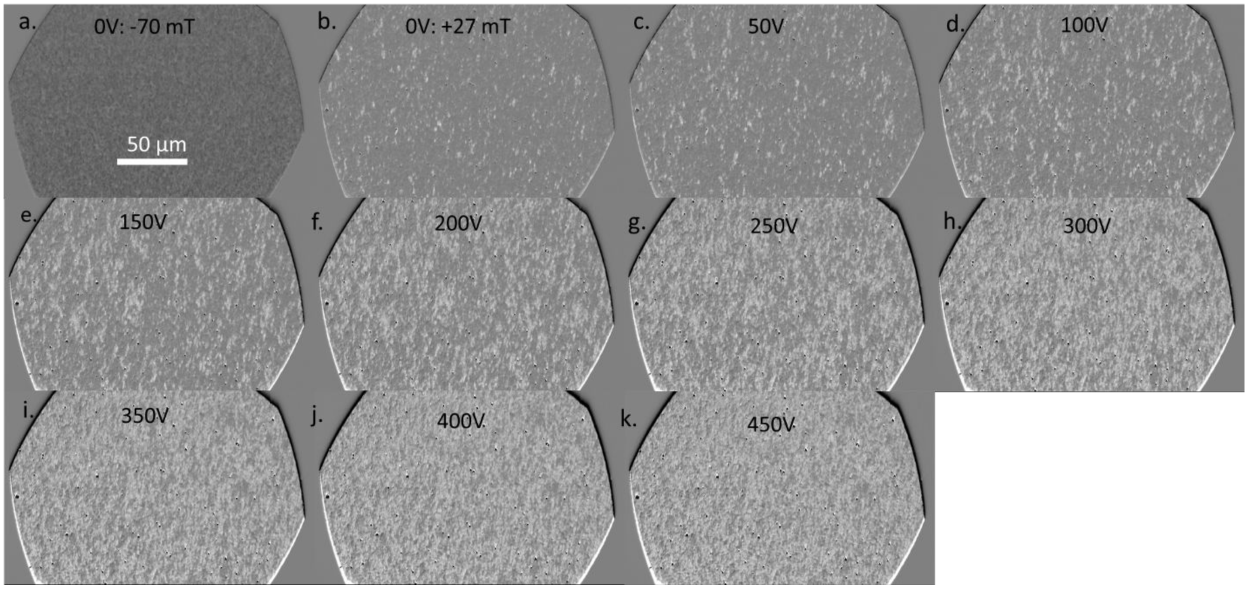

Fig. S1: MOKE images showing the saturated domains and reversal of the domains for varying amplitude voltages at a fixed reversal field along the in-plane direction $\hat{y}$ // [$01\bar{1}$]. a. The sample is poled at 450 V and relaxed to 0 V and then saturated with -70 mT field. The external field is then fixed at + 27 mT, while the voltage remains at b. 0 V and increased to c. 50 V d. 100 V e. 150 V f. 200 V g. 250 V h. 300 V i. 350 V j. 400 V and k. 450 V.

S2: Ferromagnetic Resonance:

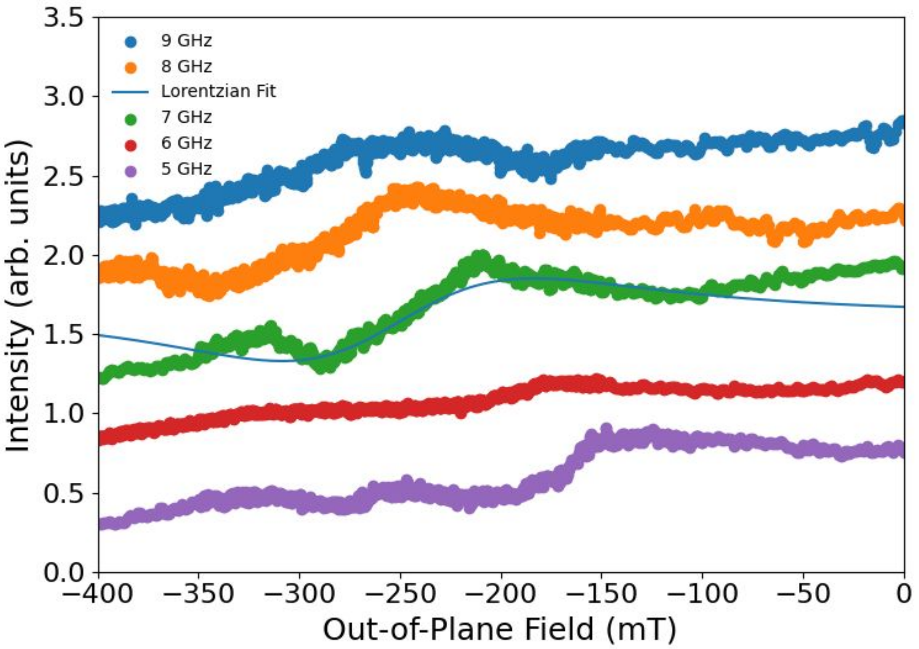

Fig. S2: FMR data for fused silica/ Bi-YIG measured at frequencies of 5 – 9 GHz, with an example of a Lorentzian fit.

S3: Estimation of Anisotropy Coefficient:

The anisotropy coefficients, $K_{eff,ij}$ (i, j =$\hat{x}$, $\hat{y}$,$\hat{z}$) are computed for different electric fields (applied voltages) using the hysteresis loops shown in Fig. 3a, 3b and 3d in the main manuscript. At first, the hysteresis loops obtained from the MOKE magnetometry are scaled with saturation magnetization, M_s to obtain the M-H loops. Next, the coefficient $K_{eff,ij}$ at a specific electric field (or the applied voltage) is estimated by computing the hysteresis loop areas (average of the descending and ascending branches) after separating the an-hysteric components [S3] for each of individual i and j directions and subsequently subtracting them. The estimated anisotropy constants for different voltages are shown in the following table SI. Uncertainty in anisotropy estimation is ~10% which is primarily coming from the uncertainty in saturation magnetization. The estimated values are rounded off to the nearest 10 J/m^3.

Table SI: anisotropy coefficients at different electric fields (applied voltages)

Voltage	$K_{eff,\hat{x}\hat{y}}$ J/m^3	$K_{eff,\hat{x}\hat{z}}$ J/m^3	$K_{eff,\hat{y}\hat{z}}$ J/m^3
0 V	840	-2590	-3420
50V	700	-2680	-3370
100V	490	-2810	-3300
150V	330	-2940	-3270
200V	210	-2990	-3200
250V	20	-3130	-3150
300V	-40	-3130	-3090
350V	-130	-3180	-3050
400V	-450	-3380	-2940
450V	-450	-3370	-2910

S4. Angular Dependent Hysteresis Loops:

Angular dependent hysteresis loops are measured under different voltages to demonstrate the 90-degree switching of the magnetic easy axis. A portion of the rectangular patterned regions of dimension 1500 um × 2200 um are illuminated with blue wavelength light to measure the hysteresis loops using longitudinal MOKE microscopy. At first the samples are poled at 450 V and then relaxed to 0 V. The samples are rotated at 10-degree steps starting from an initial position where the crystallographic direction $\hat{y}$//[0$1\bar{1}$] is at 0-degree angle and collinear with the external applied magnetic field, H_{ext}. The direction of H_{ext} is kept fixed throughout the measurements. Figure S3a shows representative MOKE hysteresis loops for sample angles (with respect to the applied magnetic field) of 0, 30, 60 and 90-degrees at 0 V. Similar to the magnetic responses in the continuous film in Fig. 3, at 0 V, the $\hat{y}$-direction (0-degree) is presented as the easy axis of magnetization and the $\hat{x}$-direction//[100] (90-degree) as the hard axis as seen from the much slower progression to saturation when the sample is rotated from 0-degree to 90-degree. In contrast, when the sample is subjected to 450 V, $\hat{y}$ no longer remains the easy axis and $\hat{x}$ becomes the easy axis. This is clear from the opposite trend of the hysteresis loops in Fig. S3b as the sample is rotated from 0-degree to 90-degree. At 450 V, the magnetization saturates much faster at 90-degree sample rotation

(when $\hat{x}$ is collinear with H_{ext}) compared to 0-degree (when $\hat{y}$- is collinear with H_{ext}). Thus a 90-degree switching of magnetic easy axis is accomplished by increasing the voltage from 0 V to 450 V.

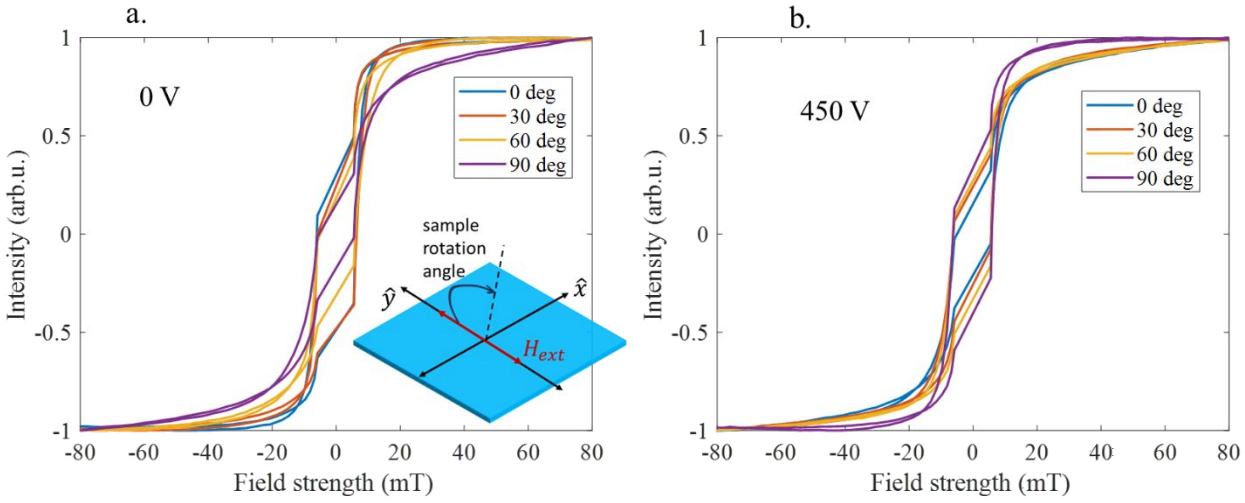

Fig. S3: Angular dependent hysteresis loops measured by longitudinal MOKE microscopy of a rectangular patterned region at voltages of a. 0 V and b. 450 V. The samples are rotated at different angles while the applied external magnetic field, H_{ext} directions are kept fixed. At 0 V, the $\hat{y}$-direction (0-degree sample angle) is the easy axis of magnetization and the $\hat{x}$-direction (90-degree) is the hard axis, and vice versa as the voltage is increased to 450 V. In figure S3, the odd shape of the loops is due to the field step size being quite large between +10 mT and -10 mT.

S5. Bi substituted YIG (Bi-YIG):

A number of possible phases exist including the orthoferrite (Bi,Y)FeO3, the garnet, and binary oxides and we need to select conditions that yield the garnet phase. YIG can be formed directly by deposition at high temperatures so can be made by a single step process. However, Bi substitution destabilizes the garnet structure (the garnet Bi3Fe5O12 is thermodynamically unstable) because of the large size of the Bi ion. When Bi-YIG is deposited at high temperatures (~560˚C) onto substrates other than garnet, we find that it forms a non-garnet phase. To illustrate this, Fig. S4a is the XRD pattern of a Bi-YIG film deposited on Si at high temperature (temperature setpoint 700 ˚C, actual temperature ~560 ˚C). The film lacks the characteristic garnet peaks seen in Fig. 1a.

Thus, a two-step process is used where amorphous Bi-YIG is deposited at room temperature and then converted to the garnet phase by annealing. This process works for films greater than a minimum thickness. A full description is given in Ref. 46 in main manuscript.

Bi-YIG is well studied in bulk and thin film form, and the Bi substitutes into the dodecahedral sites due to its large size (Shannon radii: 8-coordinated Bi3+ = 1.17Å, Y3+ =1.02Å, vs. 6-coordinated low spin Fe3+ = 0.55Å). Data such as cross-sectional HAADF TEM is consistent with dodecahedral site occupancy (the brighter atom columns indicate location of high atomic number Y and Bi) [e.g. Fig. S4b and ref. S4].

Considering the as-deposited BiYFeO film prior to crystallization, we cannot rule out depletion of Bi in the garnet compared to the target composition due to volatility of the Bi. However, a large Bi deficiency would

lead to excess Fe and the formation of Fe oxides on crystallization, which we did not observe in the film. The low temperature of deposition would also minimize Bi loss. Therefore, we do not expect that Bi is depleted in the as-deposited film.

Surface observations (AFM, SEM, EBSD) from our previous Bi-YIG study (Ref. 46 and others) did not reveal any surface phases or Bi grain boundary precipitation on Bi-YIG films crystalized at 600 ˚C. Furthermore, our previous research has shown that the garnet structure of Bi-YIG becomes unstable for crystallization at 750 ˚C and above, i.e. other phases form instead, which we attribute to Bi evaporation. Therefore, for the samples annealed at 600 ˚C in this study, we do not have evidence for significant loss of Bi.

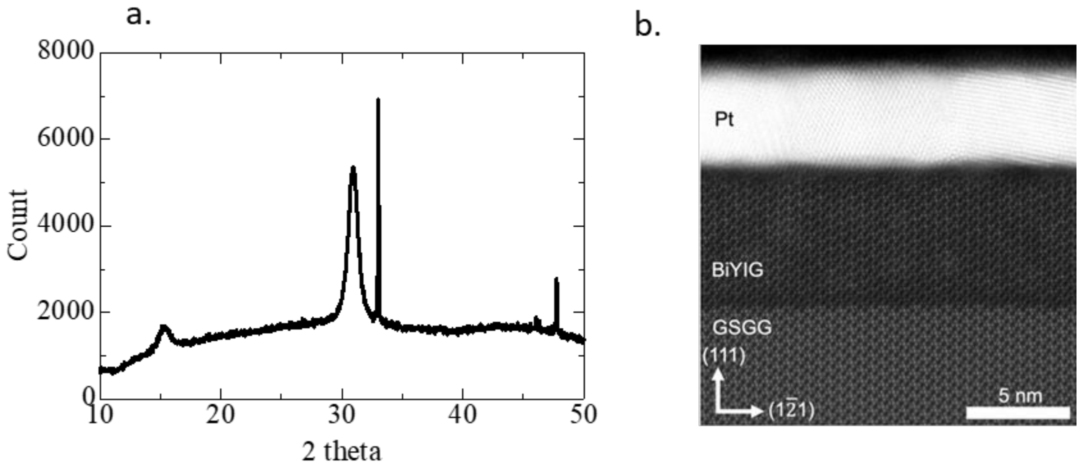

Fig. S4: a. XRD pattern of a Bi-YIG film deposited on Si at 560 ˚C. The sharp peaks are from the Si b. High angle annular dark field image (HAADF) of BiYIG on GSGG. The brighter atom columns indicate the higher Z atoms. While not quantitative, this is consistent with dodecahedral site occupancy of Bi. (TEM image courtesy of Zhen Chen, David A. Muller, Cornell University)

References:

[S1]. T. Wu, P. Zhao, M. Bao, A. Bur, J. L. Hockel, K. Wong, K. P. Mohanchandra, C. S. Lynch, and G. P. Carman, Giant electric-field-induced reversible and permanent magnetization reorientation on magnetoelectric Ni/(011)[Pb (Mg1/3Nb2/3) O3](1− x)–[PbTiO3] x heterostructure, J. Appl. Phys. 109, 124101 (2011).

[S2]. M. J. Gross, W. A. Misba, K. Hayashi, D. Bhattacharya, D. B. Gopman, J. Atulasimha, C. A. Ross, Voltage modulated magnetic anisotropy of rare earth iron garnet thin films on a piezoelectric substrate, Appl. Phys. Lett. 121, 252401 (2022)

[S3]. R. C. O'Handley, Modern Magnetic Materials: Principles and Applications,1st ed. (John Wiley & Sons, Inc., New York, NY, 1999)

[S4]. E. Rosenberg, J. Bauer, E. Cho, A. Kumar, J. Pelliciari, C. A. Occhialini, S. Ning, A. Kaczmarek, R. Rosenberg, J. W. Freeland, Y.-C. Chen, J.-P. Wang, J. LeBeau, R. Comin, F. M. F. de Groot, C. A. Ross, Revealing Site Occupancy in a Complex Oxide: Terbium Iron Garnet, Small, vol. 19, 2300824 (2023)
